# Study on the Mechanical Behavior of a Dual-Density Hybrid Lattice Structure under Quasi-Static and Dynamic Compressions

**DOI:** 10.3390/ma16103822

**Published:** 2023-05-18

**Authors:** Xinyu Li, Jiajian Ye, Yuanyuan Ding, Guoyi Wu

**Affiliations:** 1 Key Laboratory of Impact and Safety Engineering, Ministry of Education, Ningbo University, Ningbo 315211, China; 2 Ningbo Amico Copper Valves Manufacturing Inc., Ningbo 315211, China

**Keywords:** lattice structure, dual-density, energy absorption, deformation mode, strain rate effect

## Abstract

The dual-phase lattice structure composed of the matrix phase (MP) and the reinforcement phase (RP) is a novel hybrid lattice showing excellent energy absorption ability. However, the mechanical behavior of the dual-phase lattice structure under dynamic compression and the enhancement mechanism of the reinforcement phase have not been widely studied with the increase in compression speed. Based on the design requirements of dual-phase lattice materials, this paper combined octet-truss cell structures with different porosities, and the dual-density hybrid lattice specimens were fabricated via the fused deposition modeling technique. Under quasi-static and dynamic compressive loadings, the stress–strain behavior, energy absorption capacity, and deformation mechanism of the dual-density hybrid lattice structure were studied. The results showed that the quasi-static-specific energy absorption of the dual-density hybrid lattice structure was significantly higher than that of the single-density Octet lattice, and with the increase in compression strain rate, the effective specific energy absorption of the dual-density hybrid lattice structure also increased. The deformation mechanism of the dual-density hybrid lattice was also analyzed, and the deformation mode changed from an inclined deformation band to a horizontal deformation band when the strain rate changed from 10^−3^ s^−1^ to 100 s^−1^.

## 1. Introduction

With the development of aerospace and deep-sea exploration, biomedicine, and other fields, as well as the increasingly extreme working environment, the demand for high-performance, lightweight materials and structures has increased [1,2,3,4,5,6]. Lattice structures composed of regular cell structures are proven to have excellent mechanical properties and are widely used in a lot of fields [7,8]. Compared with disordered, porous materials, such as foam materials [1], lattice structures have great design space and can fully meet the requirements of high performance. By regulating the cell structure, lattice structures can exhibit some metamaterial characteristics, such as negative Poisson’s ratio [9], excellent damping characteristics [10], controllable stiffness [9,11], sound absorption and noise reduction functions [12], and double-negative refractive index [13].

The mechanical properties of lattice structures are mainly determined by the geometric characteristics of cell structures. Ashby et al. [14] designed a basic lattice structure with high strength based on the relationship between the deformation mode and the strength of the foam materials and laid an important theoretical foundation for lattice structure and mechanical designs. Mullen et al. [15] changed the geometric parameters, such as the diameter and length of the single-cell rod, and obtained the octahedral titanium alloy lattice structure, and bone in-growth surfaces exhibiting properties comparable to those of human bone were manufactured. The results showed that the compressive strength of the lattice was positively correlated with its relative density. Yan et al. [16] designed periodic cellular lattice structures by repeating a unit cell type called gyroid, which consisted of circular struts and a spherical core, and discussed the influence of cell structure size on the compression characteristics of lattices. Ravari et al. [17] established a finite element model to predict the effect of variation in the struts’ diameter on the elastic modulus, as well as the collapse stress of lattice structures. Zheng et al. [8] found that a stretch-dominated octet-truss unit cell could also ensure the constant stiffness of unit mass density at an ultra-low density; this structure had ultra-high mechanical properties in the density range of more than three orders of magnitude and was independent of the constituent materials. The mechanical properties of the lattice structure are not only related to the element structure but also affected by the arrangement and combination of its structure. Xu [18] used 3D printing technology to design four octahedral lattice structures with different relative densities and carried out ultraviolet curing treatment. Under the condition of the same relative densities, the compressive strength and compression modulus of the cured octahedral lattice structures increased significantly. The specific energy absorption increased with the increase in relative density, and the increase trend was obviously higher than that without curing the octahedral lattice structure. Yu et al. [19] proposed a lattice structure capable of stably building a three-period minimal surface with complex contour by using a three-period minimal surface and a voxel distance field. This lattice structure with a three-period minimal surface had good connectivity, controllable topology structure, was lightweight and high-strength, and has been widely used in the design of biological implants and lightweight structures.

According to the special internal combination of natural materials, such as bamboo (consisting of the hard and soft phases of the hierarchical structure), a dual-phase lattice structure composed of the matrix phase (MP) and the reinforcement phase (RP) was proposed. Yin et al. [20] designed a dual-phase hybrid lattice, with a face-centered cube (FCC) as the matrix phase and a body-centered cube (BCC) as the reinforcement phase. Through quasi-static compression tests and simulations, it was found that the addition of reinforced phase particles could improve the strength and toughness of the dual-phase hybrid lattice. A novel hybrid lattice design strategy where each matrix phase is surrounded by the reinforcement phase was proposed and exhibited excellent quasi-static energy absorption capacity, compared with that of the matrix-phase lattice. Yu et al. [21] proposed a heterogeneous strategy based on deep learning to design a heterogeneous lattice structure that consisted of rhombic dodecahedrons (RD) and octet-truss (OT) unit cells with a customized target response. The mechanical properties of the heterogeneous lattice structure were determined by a quasi-static compression test and finite element analysis. The finite element model was used to calculate the nominal stress–strain curve of the independent heterogeneous lattice structure. Based on these data, the artificial neural network was trained, verified, and tested, and the heterogeneous lattice structure with various specific target performances was successfully realized. Xiao et al. [22] designed a dual-phase hybrid lattice structure with the bending-dominated, body-centered cube structure (BCC) as the matrix phase and the tension-dominated octagonal truss structure (Octet) as the reinforcement phase. The specific stiffness of the structure was significantly improved both in transverse and longitudinal compressions. Kladovasilakis et al. [23] developed four distinct hybrid cellular materials, and the results showed that the hybrid-architected materials exhibited a superior mechanical response in comparison with their origin cellular materials. Lei et al. [24] constructed three hybrid arrangements (horizontal, vertical, and circular) composed of any two types of four typical lattice unit cells according to natural biology. The results revealed significant effects of spatial arrangement patterns and cell performance differences on the overall mechanical performances, especially in terms of ultimate strength. However, it was not difficult to find that the mechanical behavior of the dual-phase lattice structure was studied basically under quasi-static loading and rarely involved dynamic mechanical behavior, which made the application of the dual-phase lattice structure under large impact have no strong reference basis.

In this paper, a dual-phase lattice structure was selected to carry out compression tests under quasi-static and dynamic conditions, and its deformation modes at different strain rates under dynamic conditions were compared and analyzed accordingly.

## 2. Design and Fabrication of Dual-Density Hybrid Lattice Structure

### 2.1. Model Design

**Figure 1:**
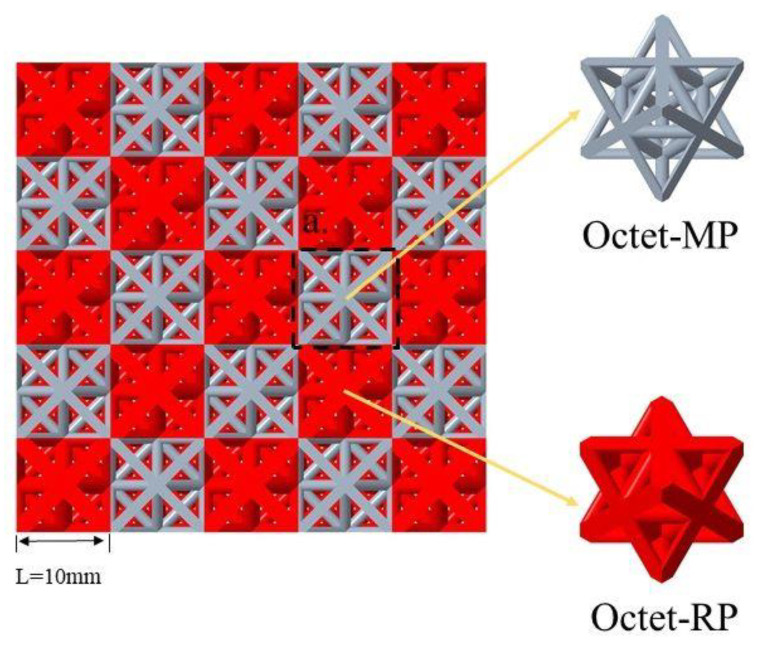
The DDHL with the largest interface area between Octet-RP and Octet-MP cell structures.

A double-density hybrid lattice (DDHL), inspired by the dual-phase mechanical metamaterial composites [20], was developed and used to study its dynamic behavior and the corresponding deformation mechanism. The modeling was carried out using the Creo Parametric software, as shown in Figure 1; two octet-truss structures [25,26,27,28,29] with different relative densities, which possess excellent stiffness and energy absorption properties as typical tension-dominated lattice structures, were used as the basic hybrid cells in the DDHL, in which the octet-truss with the small relative density was set as the matrix phase (Octet-MP), and the octet-truss with the large relative density was used as the reinforcement phase (Octet-RP). Yin et al. [20] pointed out that under the premise of the same number of reinforcement phases, the mechanical properties of the dual-phase structure under quasi-static state significantly improved with the increase in the interface area between the reinforcement phase and the matrix phase. Therefore, a double-density hybrid lattice (DDHL) with a maximum interface area between the reinforcement phase and the matrix phase was proposed, where each matrix phase was completely surrounded by the reinforcement phase, as shown in Figure 1.

### 2.2. Sample Preparation

**Figure 2:**
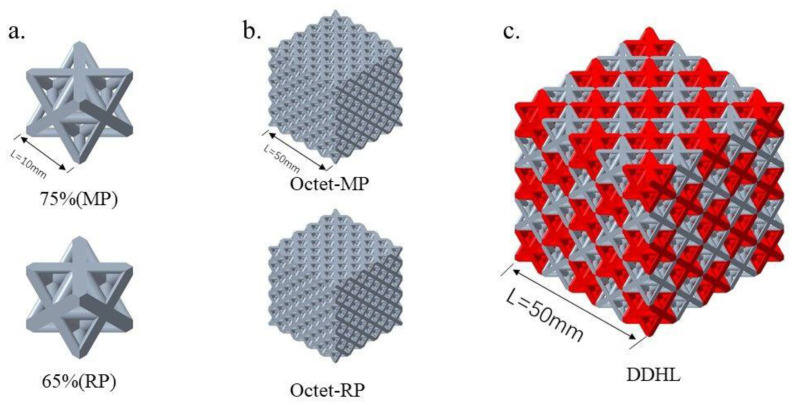
(**a**) Octet-MP and Octet-RP cell structures; (**b**) Octet lattice model; (**c**) the DDHL structure (red is the reinforcement phase).

Considering that the diameter of the strut rod in the Octet cell structure will be very small when its porosity is too large, resulting in structure loss during 3D printing, in the process of sample preparation, the porosities of Octet-MP and Octet-RP cell structures were set to 65% and 75%, respectively, corresponding to strut diameters of 1.9 mm and 1.56 mm. Further considering the reliability of 3D printing and the stability of the hybrid lattice structure, the DDHL structure was composed of 5 × 5 × 5 cell units along the three-dimensional orthogonal direction. The overall size of the Octet structure was 50 × 50 × 50 mm^3^, and the size of each Octet cell structure was 10 × 10 × 10 mm^3^. To ensure the largest contact area between Octet-RP and Octet-MP cell structures in the DDHL, the matrix phase and the reinforcement phase were arranged one by one; that is, all six surfaces of each Octet-RP were in contact with the Octet-MP, as shown in Figure 2c. Thus, the dual-density hybrid lattice (DDHL) structure included 63 reinforced single-cell structures and 62 matrix single-cell structures. At the same time, single-density lattices with Octet-RP or Octet-MP were also established to compare the mechanical behaviors of the DDHL, as shown in Figure 2b.

**Figure 3:**
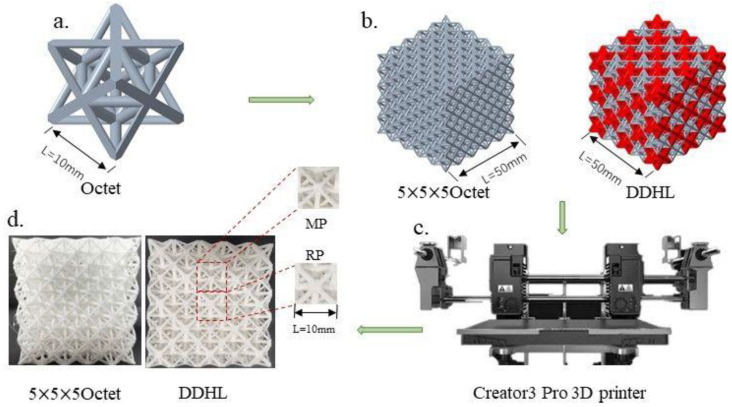
The fabrication process of the Octet and the DDHL specimens: (**a**) Octet cell structure; (**b**) Octet lattice and DDHL structure models; (**c**) 3D printing equipment; (**d**) Octet lattice and DDHL specimens.

Due to the complexity of the designed hybrid lattice, additive manufacturing technology was used to process the test specimens required for the experiments, and the fabrication process is shown in Figure 3. According to different 3D printing process characteristics, such as selective laser melting [30], fused deposition modeling, and stereo lithography apparatus [31,32], the fused deposition modeling (FDM) had the advantages of fast printing, low manufacturing cost, and low printing requirements. Thus, the DDHL and pure Octet lattice specimens were fabricated by fused deposition modeling (FDM). PolyMax Polylactide (PLA) filaments (produced by the Shanghai Ploymaker company), which possess high toughness and durability, were employed as the constituent material, and its physical properties and tensile mechanical behavior are shown in Table 1, provided by the manufacturer. The melting temperature of the filamentous PLA material was 149 °C; however, the printing temperature was set at 190–230 °C during printing so that PLA would be sprayed from the nozzle after being heated and melted inside the nozzle. By controlling the nozzle to extrude the material at the specified position, the melted PLA material was continuously deposited on the base panel or the previous layer of solidified material, and the temperature of the printing base panel was kept at 25–60 °C so that the PLA material could be quickly solidified. During the fabrication, the printing nozzle with a diameter of 0.4 mm moved at the speed of 50 mm/s in a circular printing path for each layer, and finally, the Octet lattice and the DDHL specimens are shown in Figure 3d. The actual sizes of the lattice samples are shown in Table 2. Compared with the designed model, the maximum difference in porosity was only 1.3%, which indicates that the precision of 3D printing was very good.

## 3. Quasi-Static and Dynamic Compression Tests of Octet Lattice and DDHL

### 3.1. Quasi-Static Behavior of Octet Lattice and DDHL Structures

**Figure 4:**
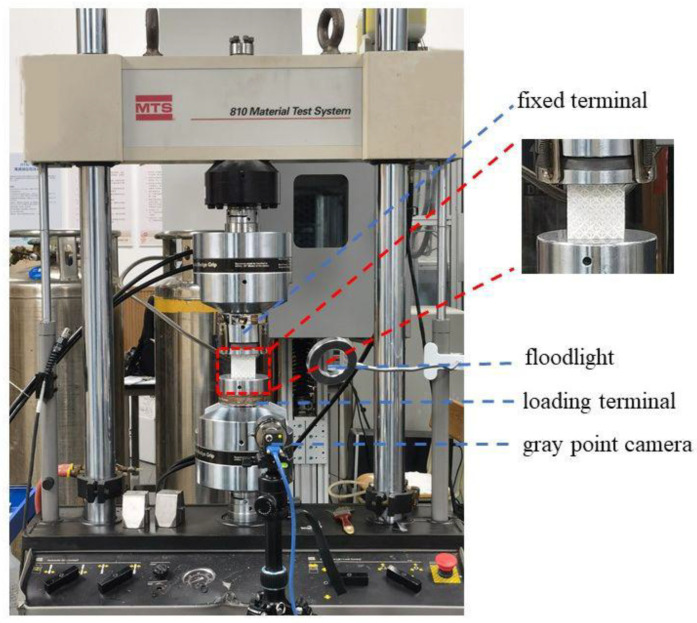
Quasi-static compression experimental device.

To characterize the overall engineering quasi-static stress–strain responses and energy absorption characteristics of DDHL structures, a MTS810 universal testing machine, which was manufactured by MTS Systems Corporation (the company address: Eden Prairie, MN 55344, USA), was employed for uniaxial compression tests. During compression, the lattice specimens were sandwiched between two platens, where the lower platen moved upward at a constant speed of 0.05 mm/s, and the upper platen was fixed. Thus, the corresponding global engineering strain rate was controlled at 10^−3^ s^−1^. The interfaces between a lattice sample and two compressing platens were lubricated with grease to minimize friction. At the same time, the deformation process and failure mode of the specimen were recorded with a gray point camera. The experimental device is shown in Figure 4. Three repeated experiments were carried out for each lattice structure to ensure the reliability of the results.

**Figure 5:**
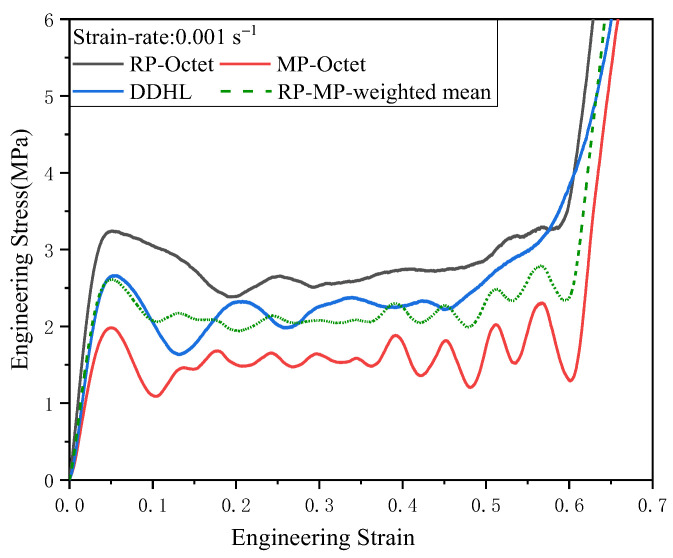
Engineering stress–strain curve of the Octet structure and the DDHL structure under the uniaxial compression test.

The quasi-static response of the DDHL was compared with that of the Octet lattices to investigate the energy absorption performance and deformation mechanism of the DDHL. The engineering stress–strain curves of the Octet lattice and DDHL specimens are shown in Figure 5. Typically, the stress–strain curves of the Octet lattice and the DDHL exhibit three stages: an elastic stage and a plateau stage, followed by a densification stage. The engineering stress–strain curve of the DDHL was basically between the engineering stress–strain curves of RP-Octet and MP-Octet lattices, and the initial peak stress of the DDHL was higher than the average initial peak stress of RP-Octet and MP-Octet (which was calculated according to the proportion of the number of cells, as shown by the dotted line in Figure 5). In the plateau phase, the stress–strain curve of the DDHL was higher than the weighted average stress–strain curve of RP-Octet and MP-Octet. Thus, the mechanical properties of the dual-phase structure were not the simple addition of the mechanical properties of the matrix phase and the reinforcement phase. Although RP-Octet and MP-Octet lattices were the same geometric configurations, the fluctuation of MP-Octet was more pronounced than that of RP-Octet, due to the different diameters of its struts and the printing defects caused by 3D printing, and the DDHL structure effectively avoided this fluctuation. Observing the densification stage of the MP-Octet and RP-Octet lattices, there was an obvious turning point from the plateau stage to the densification stage. However, there was no particular change point from the plateau stage to the densification stage in the DDHL structure, and the stress–strain curve started to rise slowly from plateau stage at *ε* = 0.45 smoothly to the densification stage, which was because the Octet lattice collapsed layer by layer. Thus, the densification turning point of the Octet lattice occurred when the last layer completely collapsed. The DDHL exhibited a different deformation mode without an apparent layer-by-layer collapse.

**Figure 6:**
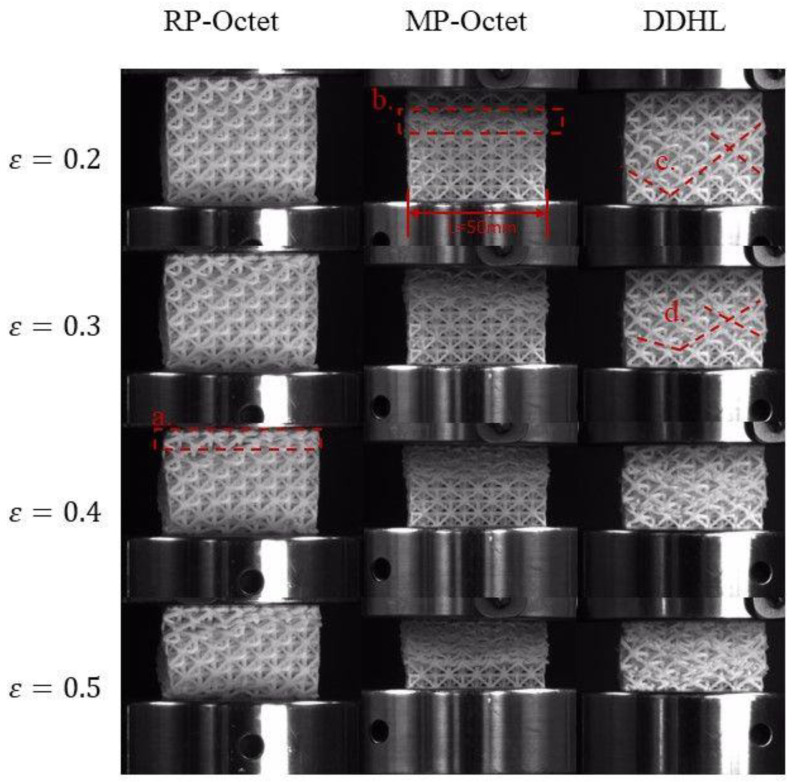
Comparison of the deformation processes between the Octet structure and the DDHL structure.

As the engineering stress–strain response of the DDHL differed markedly from that of the Octet lattice, the deformation patterns of the DDHL and the Octet lattice were recorded by a high-speed camera and compared with each other, as shown in Figure 6. However, Octet lattices with different relative densities exhibited distinct modes of collapse during deformation. The MP-Octet lattice underwent layer-by layer deformation, which led to several fluctuations in the corresponding stress–strain curve (Figure 5). In contrast, the RP-Octet lattice initially experienced localized buckling, followed by uniform deformation, resulting in a smooth evolution of the quasi-static compressive curve with an initial stress oscillation. The DDHL exhibited a dramatic dissimilarity in its deformation behavior, with the matrix phase preferentially deforming and the reinforcement phase gradually deforming with increasing strain. When the compressive strain was *ε* = 0.2, a linear collapse shear band was initially formed, due to the deformation of the matrix phase, as shown in c in Figure 6c. Similarly, with the continuous increase in strain, when *ε* = 0.3, it was no longer a single shear band but several shear bands that were connected, as shown in Figure 6d, and the shear band gradually became invisible, due to the gradual deformation of the enhanced phase with the compression process. Thus, the deformation mode of the DDHL was determined by the combination of the matrix phase and the reinforcement phase. The weaker matrix phase deformed first, and then the reinforcement phase deformed again. Therefore, interfacial sliding between the matrix phase and the reinforcement phase brought additional forces, which could be used to explain why the stress–strain curve of the DDHL was generally higher than the average stress–strain curve of MP-Octet and RP-Octet lattices.

### 3.2. Comparison of Specific Energy Absorption between Octet Lattice and DDHL Structures

The energy absorption capacity is an important index to evaluate the mechanical properties of materials in engineering applications [33,34], and the energy absorption of porous materials is closely related to the relative density. For the Octet lattice, the energy absorption was mainly the energy dissipated by the bending and fracturing of the struts, while for the DDHL structure, due to the different porosities of the matrix phase and the reinforcement phase, besides the energy dissipated by the deformation of the cell structures, the interfacial sliding between the matrix phase and the reinforcement phase produced additional energy dissipation. To study the energy dissipation caused by the interfacial sliding and to eliminate the influence of porosity on energy absorption, specific energy absorption (SEA) *Q* was used to measure its energy absorption and performance, that is, the energy absorbed per unit mass [35,36,37], which can be expressed as:

(1) $$Q=\frac{\int_{0}^{\varepsilon_{x}}\sigma d\varepsilon }{\rho }$$

where *Q* is the specific energy absorption, *ρ* is the density of the specimen, *ε_X_* represents a specific compression strain, and *σ* is stress.

When the specimen was fully compacted, the stress increased sharply, and the energy absorption of the specimen also increased sharply. However, the energy absorption was useless when the specimen was compacted, since the compressive stress was very large. The effective energy absorption capacity of the specimen is usually determined by the effective compression strain, also known as the densification strain ε*_D_*, beyond which the lattice specimen can be considered a compacted solid. Densification strain ε*_D_* can be determined with the aid of energy absorption efficiency *η* [38,39,40], which can be expressed as:

(2) $$\eta =\frac{\int_{0}^{\varepsilon }\sigma \left(\varepsilon \right)d\varepsilon }{\sigma_{\max}\left(\varepsilon \right)}$$

where *σ_max_*(*ε*) is the maximum compressive stress in the interval [0, *ε*], except for the initial peak stress [35].

**Figure 7:**
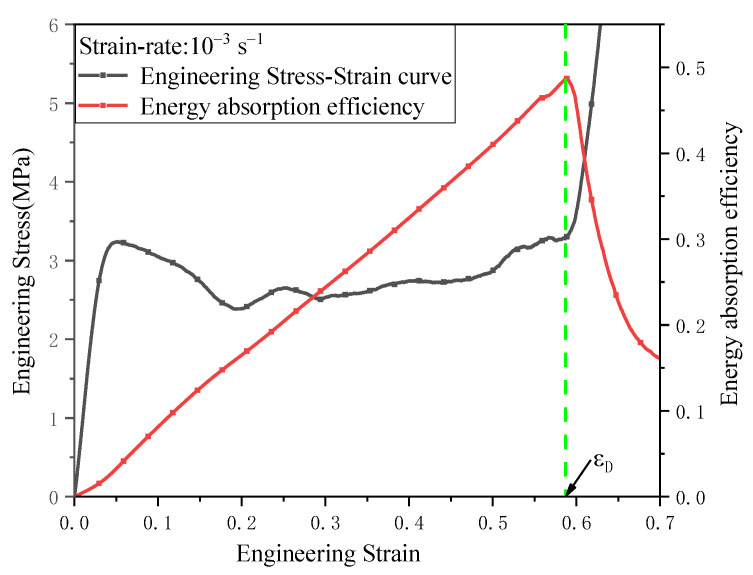
Engineering stress–strain curve and energy absorption efficiency of the Octet-RP structure under the uniaxial compression test.

With the increase in compression strain, the compression strain corresponding to the maximum energy absorption efficiency *η* was the densification strain ε*_D_*, with Octet-RP as an example, as shown in Figure 7. It was obvious that the stress increased very quickly after the densification strain was exceeded, which means that the effective energy absorption capacity of both the Octet structure and the DDHL can be estimated by the energy absorbed from 0 to *ε_D_*. Therefore, the effective specific energy absorption *Qe* can be expressed as:

(3) $$Q_{e}=\frac{\int_{0}^{\varepsilon_{D}}\sigma \left(\varepsilon \right)d\varepsilon }{\rho }$$

However, for dual-phase lattice materials, the traditional method of calculating the densification strain by determining the energy absorption efficiency was not applicable [41] because the matrix phase was deformed first, and then the reinforcement phase was deformed. Sha Yin et al. defined the calculation method of the densification strain ε*_D_* of dual-phasic lattices [20] according to the compression characteristics, which can be expressed as

(4) $$\varepsilon_{D}=\frac{n-k}{n}\left(\varepsilon_{D}^{MP}+∆\varepsilon \right)+\frac{k}{n}\varepsilon_{D}^{RP}$$

where *n* is the total number of cells, *k* is the number of reinforcement phase cells, $\Delta \varepsilon =(\sigma_{D}^{RP}-\sigma_{D}^{MP})/\psi$ represents the extra deformable strain after matrix phase densification when the stress reaches the densification stress of the reinforcement phase $\sigma_{D}^{RP}$, $\sigma_{D}^{MP}$ is the compaction stress of the reinforcement phase, ψ is the slope of the compacted section of the matrix phase, and $\varepsilon_{D}^{MP}$ and $\varepsilon_{D}^{RP}$ are the densification strains of the matrix phase and the reinforcement phase, respectively.

**Figure 8:**
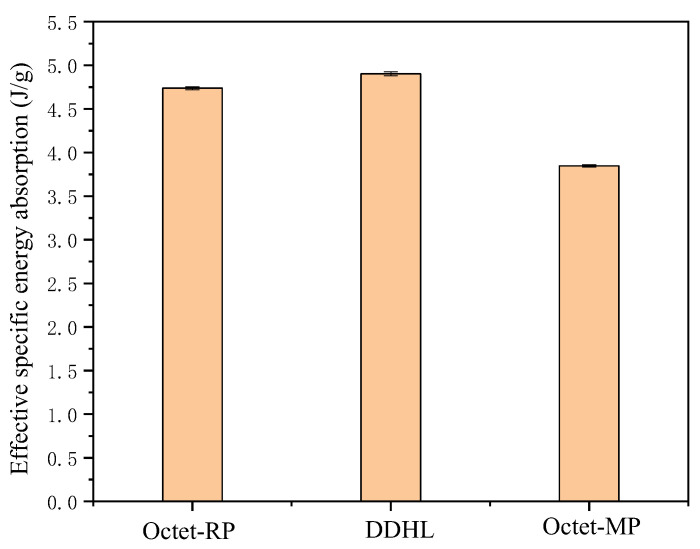
Effective specific energy absorption of the Octet structure and the DDHL structure.

According to the above equation, the effective specific absorption energy of homogeneous Octet-RP and Octet-MP structures and DDHL structures can be easily calculated, as shown in Figure 8. According to the information in Figure 8, the effective specific absorption energy of Octet-RP and Octet-MP were 4.738 J/g and 3.846 J/g, respectively, while the effective specific absorption energy of the DDHL was as high as 4.902 J/g, which was not only significantly higher than the weighted specific absorption energy of the matrix phase and the reinforcement phase at 4.300 J/g, it was also higher than the effective specific absorption energy of the Octet-RP structure. Thus, the effective specific energy absorption of the DDHL structure was not the simple addition of the mechanical properties of the matrix phase and the reinforcement phase, which is related to the special deformation mechanism of the dual-phase structure. The difference in porosity between the reinforcement phase and matrix phase led to the phase boundary slip, which increased the energy dissipation. 

### 3.3. Dynamic Experimental Results of the DDHL Structure

**Figure 9:**
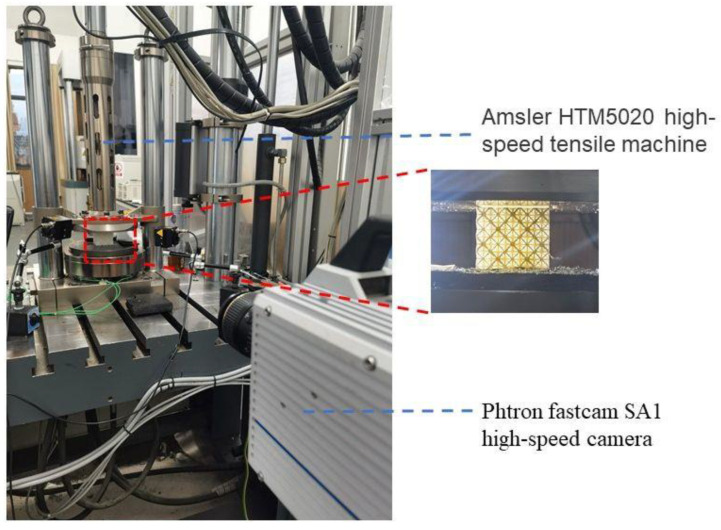
Dynamic compression experimental device.

To investigate the mechanical behavior and deformation mode of the two-phase structure under dynamic compressions, dynamic compression experiments with strain rates of 1 s^−1^, 10 s^−1^, and 100 s^−1^ were carried out using the Amsler HTM5020 high-speed tensile machine (Zwick, Germany), which enabled a constant compression/tension at loading speeds from 0.001 m/s to 20 m/s. A Photron Fastcam SA1 high-speed camera was used to record the deformation process at 4000 frames per second, with 512 × 512 pixels. During the experiment, the lower compression platform was fixed, and the upper compression platform moved downward at speeds of 50 mm/s, 500 mm/s, and 5 m/s. When the strain rate was less than or equal to 10 s^−1^, the high-speed camera was triggered by the synchronization box to ensure the synchronization of the deformation figure and the compressive data. When the strain rate was 100 s^−1^, the loading rate was fast, making it difficult to successfully trigger the synchronization box. Therefore, the manual trigger method was used to start the recording of the high-speed camera. The loading platform and the specimen placement are shown in Figure 9.

**Figure 10:**
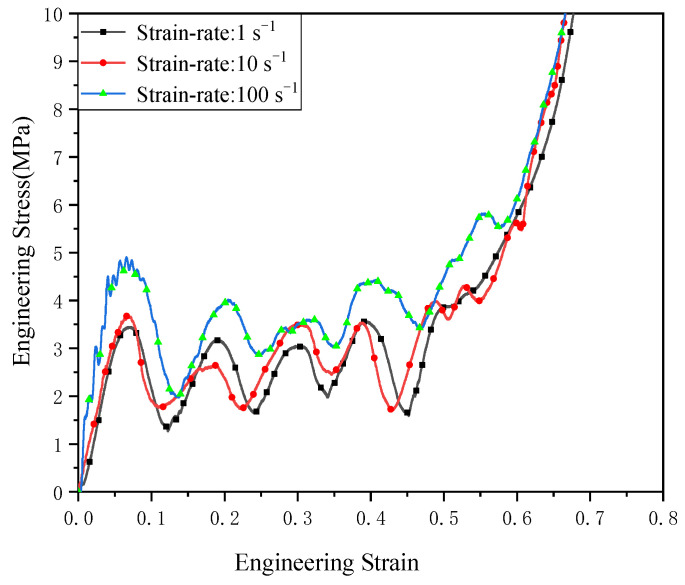
Engineering stress–strain curve of the DDHL structure under dynamic compressions.

The engineering stress–strain curves of the dynamic compression tests of the DDHL structure are shown in Figure 10. Compared with the engineering stress–strain curve under quasi-static compression, the initial peak stress of the stress–strain curve under dynamic compression increased significantly. The peak stress under quasi-static compression was about 2.66 MPa, while the dynamic initial peak stresses under strain-rates of 1 s^−1^, 10 s^−1^, and 100 s^−1^ were about 3.45 MPa, 3.67 MPa, and 4.4 MPa, respectively. In the plateau stage, compared with the quasi-static compression, the stress–strain curve showed obvious fluctuations. The inflection point from the platform segment to the compacted segment cannot be observed in the figure because the fluctuation from the platform segment to the compacted segment is very small.

**Figure 11:**
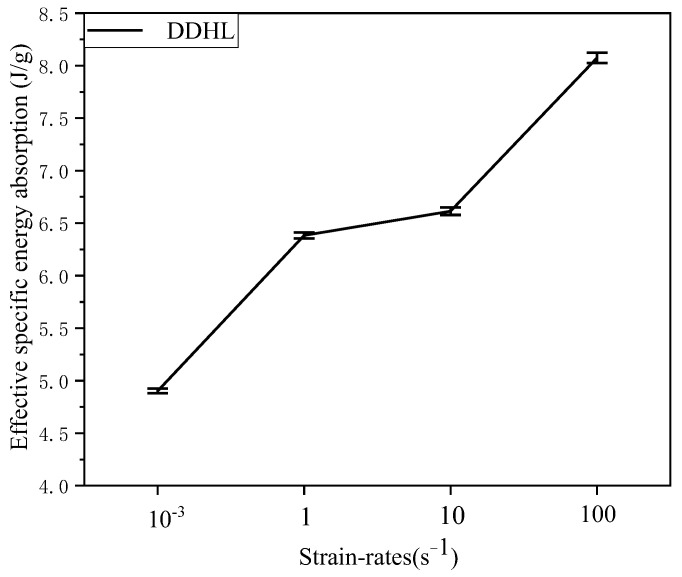
Effective specific energy absorption of the DDHL at different strain rates.

Hence, the effective specific absorption energy of the DDHL structure under different strain rates can be calculated according to Equation (4), and its change trend with the increase in strain rate is shown in Figure 11. It can be observed that the effective specific absorption energy of the DDHL structure increased significantly with the increase in the strain rate, and the effective specific absorption energy under dynamic experiment could be improved significantly, compared with the quasi-static experiment. The improvement of this property was related to the deformation mode at different strain rates.

**Figure 12:**
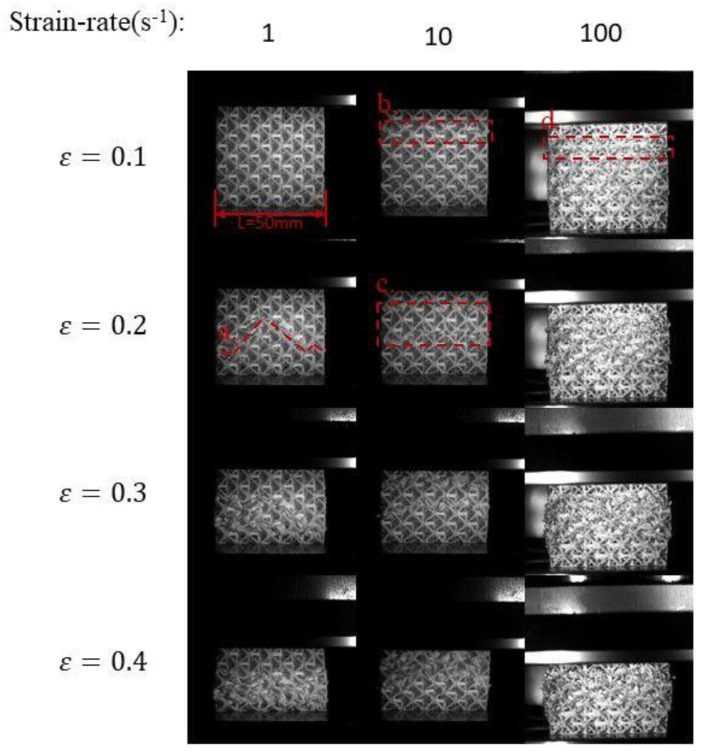
Dynamic deformation patterns of the DDHL structure.

According to the dynamic deformation patterns of the DDHL in Figure 11, it can be found that the dynamic deformation modes under strain rates of 1 s^−1^ and 10 s^−1^ had no change in essence, compared with the quasi-static deformation mode, forming a deformation zone caused by the initial crushing deformation of the matrix phase, followed by the deformation of the reinforcement phase, as shown in Figure 12a,c. However, when the strain-rate was 10 s^−1^, the deformation zone formed by the matrix phase deformation was not as clear as the deformation zone formed when the strain-rates were 10^−3^ s^−1^ and 1 s^−1^ and even showed a trend of layer-by-layer deformation, as shown in Figure 12b. When the compressive strain rate reached 100 s^−1^, it was difficult to clearly observe the inclined deformation zone formed by the deformation of the matrix phase. The deformation of the DDHL under a strain rate of 100 s^−1^ was mainly manifested as the deformation mode of layer-by-layer crushing. This was because, with the strengthening of the inertial effect, the weaker matrix phase had no time to deform, so it deformed together with the indenter and the reinforcement phase, as shown in Figure 12d.

## 4. Conclusions

In order to study the deformation mode and dynamic behavior of dual-phase hybrid lattice structures, the paper mixed two Octet lattices with different relative densities to realize the design of the dual-density hybrid lattice (DDHL) and carried out quasi-static and dynamic experiments with MTS 810 and Amsler HTM5020 high-speed tensile machines. The deformation modes of the DDHL structure under different strain-rates were observed, and the engineering stress–strain curves were obtained. The following conclusions were drawn from the analysis:

1. Compared with the Octet lattice, the specific energy absorption of the DDHL structure was significantly improved because the interfacial sliding between the matrix phase and the reinforcement phase brought additional energy dissipation.
2. With the increase in the compressive strain rate, the effective specific absorption energy of the DDHL was enhanced, due to the gradual emergence of inertial effects.
3. Under dynamic compressive loading, the deformation band initially formed by the matrix phase deformation of the DDHL structure was not obvious. Under a compressive strain rate of 100 s^−1^, the deformation mode of the DDHL exhibited a layer-by-layer collapse.

## Figures and Tables

**Table 1:** Material properties of PLA measured at 25 °C.

Property	Parameter	Numerical Value
physical property	density	1.17–1.24 (g/cm^3^)
	melting temperature	149 (°C)
	crystallization temperature	112 (°C)
mechanical behavior	modulus of elasticity	1879 ± 109 (MPa)
	elongation at break	1.4 ± 0.3 (%)

**Table 2:** Actual sample size.

Number	Long	Wide	High	Weigh	Porosity
Octet-MP	49.96	50.3	49.26	30.07	75.7%
Octet-RP	49.9	49.78	49.78	41.83	66.2%
DDHL	49.94	49.92	49.64	35.55	71.3%

## Data Availability

Data and sources available upon request from authors.
